# Docetaxel/S-1 Versus Docetaxel/Capecitabine as First-Line Treatment for Advanced Breast Cancer: A Retrospective Study

**DOI:** 10.1097/MD.0000000000001340

**Published:** 2015-10-16

**Authors:** Jinyu Li, Junhao You, Wen Si, Yanyun Zhu, Yi Chen, Bo Yang, Chun Han, Ruixia Linghu, Xingyang Zhang, Shunchang Jiao, Junlan Yang

**Affiliations:** From the Department of Medical Oncology (JL, WS, YZ, RL, XZ, SJ, JY, BY, CH), Chinese PLA General Hospital, Beijing; Department of Medical Oncology (JY), Hainan Branch of PLA General Hospital, Sanya; School of Medicine (WS), Nankai University, Tianjin; and Medical Center of Tsinghua University (YC), Beijing, China.

## Abstract

The treatment efficacy of advanced breast cancer is still not promising. This study aimed to compare the efficacy and safety of docetaxel/S-1 (DS1) versus docetaxel/capecitabine (DX) as the first-line treatment for advanced breast cancer.

From June 2008 to June 2013, 22 patients with advanced breast cancer were treated with the DS1 regimen. Another 26 age- and disease status-matched patients treated with the DX regimen served as controls. The 2 groups were compared in terms of time to progression (TTP), objective response rate, disease control rate, clinical benefit rate, and safety profiles.

Median TTP did not differ significantly between the DS1 group and the DX group (9.04 vs 10.94 months, *P* = 0.473). There were no significant differences in objective response rate, disease control rate, and clinical benefit rate between the 2 groups. Both the DS1 and the DX regimens showed good tolerability. The 2 regimens showed no significant difference in adverse events except degree III hand-foot syndrome (DS1 0 vs DX 23.1%, *P* = 0.025).

For the first-line treatment of advanced breast cancer, the DS1 and the DX regimens showed similar efficacy and safety. The DS1 regimen had less severe hand-foot syndrome than the DX regimen.

## INTRODUCTION

Breast cancer is the most common malignancy in women. About 20% to 35% patients still develop recurrence and metastasis after radical resection of the tumor.^1^ Advanced metastatic breast cancer is incurable, and treatment target of advanced breast cancer is to control the symptoms, improve quality of life, and prolong life of the patients. Although the treatment efficacy of advanced breast cancer has been greatly improved in recent years, the median survival of these patients is still only 2 to 3 years.^2,3^

Anthracyclines and taxanes are commonly used first-line treatments for advanced breast cancer. Owing to the use of anthracyclines in the adjuvant or neoadjuvant chemotherapy and its cardiotoxicity, the application of these drugs in advanced breast cancer has been limited. Advanced breast cancer patients treated with docetaxel had a response rate of 48% and a median survival of 16 months.^4,5^ Docetaxel has become a cornerstone in the treatment of advanced breast cancer. Combination chemotherapy has higher objective response rate and longer time to progression (TTP) than single agent chemotherapy in the treatment of advanced breast cancer. The gains in survival of combination chemotherapy are modest in comparison with the single agent chemotherapy.^6,7^ However, the combination chemotherapy of docetaxel and capecitabine has shown significantly improved response rate and overall survival than docetaxel monotherapy in treating advanced breast cancer.^8,9^ Therefore, the docetaxel/capecitabine (DX) regimen is increasingly being used in the treatment of advanced breast cancer.

S-1 (Taiho Pharmaceutical Co., Tokyo, Japan) is an oral fluoropyrimidine derivative composed of 1-(2-tetrahydrofuryl)-5-fluorouracil (tegafur, a prodrug of 5-flurouracil [5-FU]), combined with 2 modulators of 5-FU activity, 5-chloro-2, 4-dihydroxypyrimidine (gimeracil) and potassium oxonate (oteracil). Gimeracil is a dihydropyrimidine dehydrogenase inhibitor, and helps to decrease 5-FU catabolism and increase the blood levels of 5-FU. Oteracil potassium, another enzyme inhibitor of 5-FU, can suppress the gastrointestinal toxicity of tegafur.^10^ S-1 and capecitabine have similar pharmaceutical mechanisms by converting to the anticancer drug of 5-FU. Treatment of advanced breast cancer using S-1 alone has a response rate of 41.7%.^11^ However, there is no report on the efficacy and safety of combination therapy of docetaxel and S-1 in the treatment of advanced breast cancer.

We performed a retrospective study to compare the efficacy and safety of the docetaxel/S-1 (DS1) regimen and the DX regimen in treating advanced breast cancer.

## MATERIALS AND METHODS

### Patients

This retrospective study included 22 patients with advanced breast cancer treated with the DS1 regimen as the first-line treatment from June 2008 to June 2013 at our hospital. Another 26 advanced breast cancer patients treated with the DX regimen as the first-line treatment served as controls. The DX group was selected by matching to the DS1 group in age, metastatic site, Karnofsky performance score (KPS), ER/PR/HER2 status, and history of chemotherapy. The inclusion criteria were as follows: age ≥18 years; definite pathological diagnosis; untreated metastatic invasive ductal carcinoma; KPS 80 to 100 with expected survival of 3 months; with evaluable lesions; and if taxanes were used in neoadjuvant or adjuvant therapy, at least 12 months were required from the end of the treatment to the recurrence. This study was approved by the Institutional Review Board of General Hospital of Chinese PLA. Each of the 22 patients has signed a written informed consent form. All procedures performed in studies involving human participants were in accordance with the ethical standards of the institutional and/or national research committee and with the 1964 Helsinki declaration and its later amendments or comparable ethical standards.

### Chemotherapy Regimens

The DS1 regimen included docetaxel on day 1 (75 mg/m^2^, IV drop for 1 hour) and S-1 capsules for day 1 to 14 (40 mg/m^2^, oral, bid). The DX regimen included docetaxel on day 1 (75 mg/m^2^, IV drop for 1 hour) and capecitabine (1000 mg/m^2^, oral, bid).

### Outcome Evaluation

Treatment efficacy was evaluated according to the Response Evaluation Criteria in Solid Tumors and classified into complete response (CR), partial response (PR), stable disease (SD), and progressive disease (PD). Adverse events were evaluated using the National Cancer Institute Common Toxicity Criteria version 3.0 and classified as degree 0 (none), degree I (mild), degree II (moderate), degree III (severe), and degree IV (life-threatening).

The primary outcome of our study was the TTP. TTP was defined as the time from treatment initiation to disease progression, or death caused by tumor or other reasons, or the last follow-up of patients not showing disease progression. The secondary outcomes were objective response rate, disease control rate, and clinical benefit rate. Objective response rate was the sum of CR and PR. Disease control rate was calculated by adding up CR, PR, and SD. Clinical benefit rate was defined as the sum of CR, PR, and SD (≥6 months).

### Statistical Analysis

Continuous data were expressed as medians. Numerical data were expressed as frequencies or percentages and compared using χ^2^ test. Survival was analyzed using Kaplan–Meier analysis and compared using log-rank test. *P* < 0.05 was considered statistically significant.

## RESULTS

### Patient Demographics

No significant difference was found in age, KPS, and disease characteristics between the DS1 group and the DX group (Table 1). Patients were followed up every 6 months. The median follow-up time was 10 months in the DS1 group and 15 months in the DX group.

**TABLE 1:**
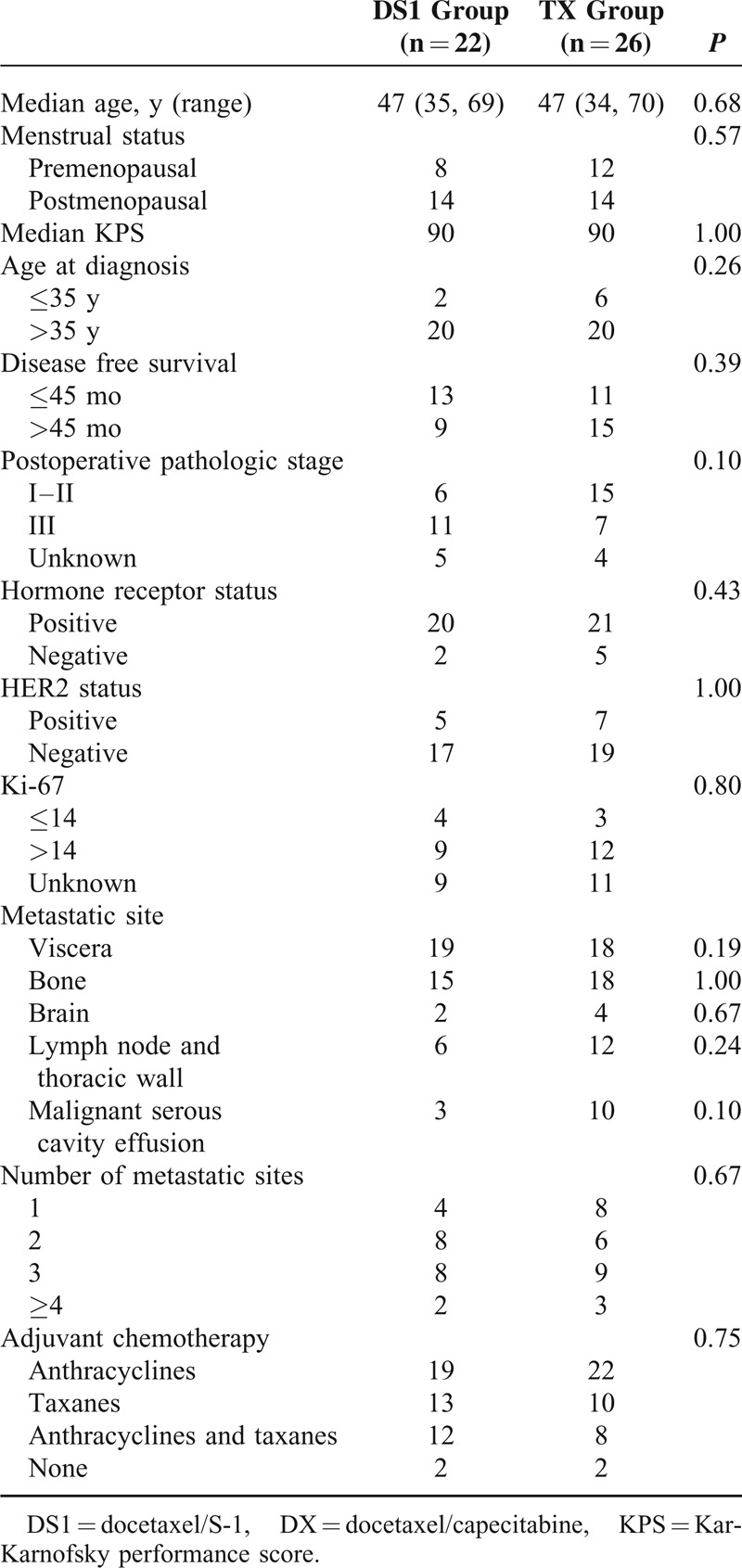
Demographic Information of the DS1 Group and the TX Group

### Chemotherapy Details

The DS1 group received a median of 6 cycles of chemotherapy (range, 4–8 cycles), and the DX group received a median of 6 cycles of chemotherapy (range, 2–8 cycles). In the DS1 group, 14 patients did not develop PD after 4 to 8 cycles of chemotherapy. Of these patients, 11 patients received sequential S-1 maintenance therapy (78.6%, 11/14), 2 patients received hormone maintenance therapy (14.3%, 2/14), and 1 patient gave up treatment (7.1%, 1/14). In the DX group, 20 patients did not develop PD after 2 to 8 cycles of chemotherapy. Of these patients, 15 patients received sequential capecitabine maintenance therapy (75%, 15/20), 4 patients received hormone maintenance therapy (20%, 4/20), and 1 patient received docetaxel maintenance therapy (5%, 1/20). Therefore, the treatments of the DS1 group and the DX group were similar.

### Treatment Efficacy

**FIGURE 1:**
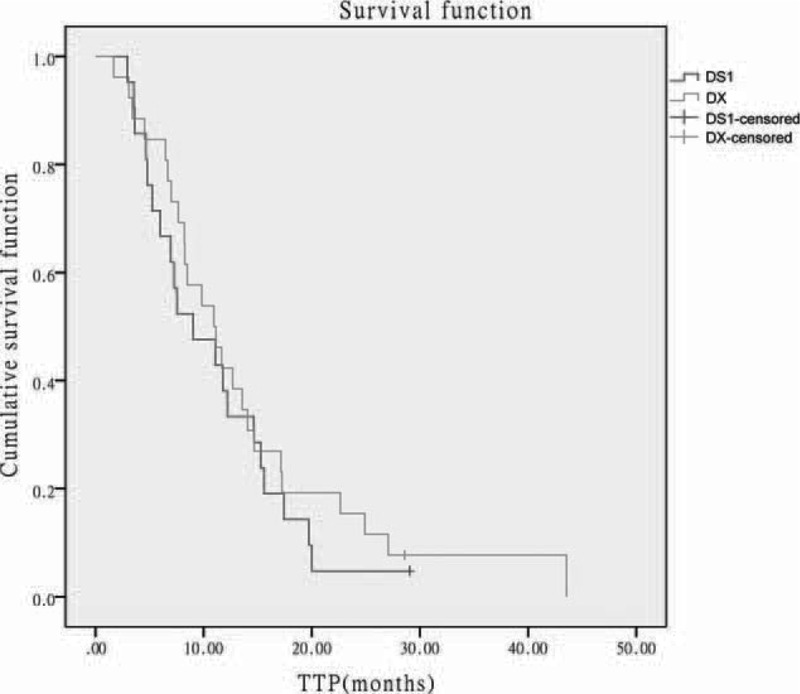
Comparison of time to progression between the DS1 group and the DX group. DS1 = docetaxel/S-1, DX = docetaxel/capecitabine.

The DS1 group and the DX group did not differ significantly in TTP (DS1: median 9.04 months, 95% confidence interval [CI] 3.34–14.73 months; DX: median 10.94 months, 95% CI 6.96–14.92 months; *P* = 0.473; hazard ratio [HR] = 0.852, 95% CI 0.441–1.462) (Fig. 1). There was no significant difference in objective response rate, disease control rate, and clinical benefit rate between the 2 groups (*P *> 0.05; Table 2).

**TABLE 2:**
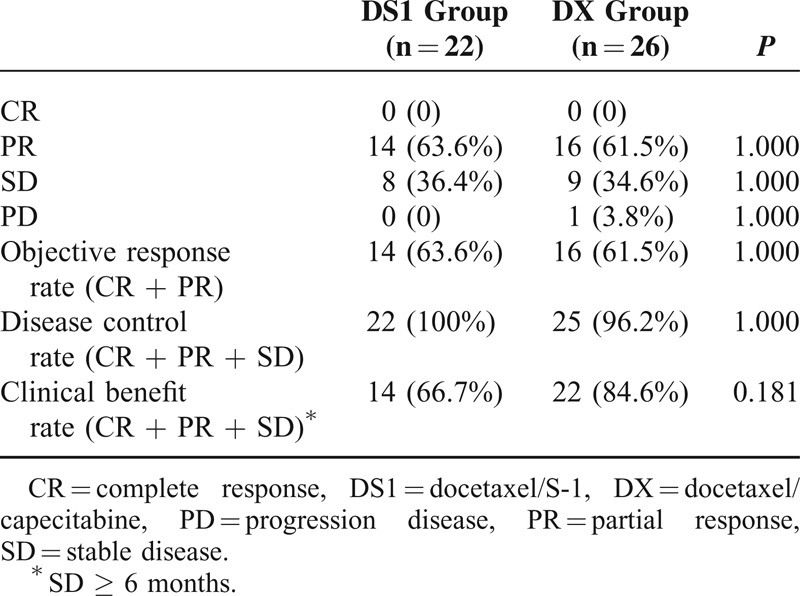
Comparison of Efficacy Between the DS1 Group and the DX Group (n, %)

### Adverse Events

The most common adverse events were gastrointestinal symptoms and leukopenia. The DS1 group and the DX group did not differ significantly in gastrointestinal symptoms (77.3% vs 80.8%, *P* = 1.000) and leukopenia (95.5% vs 88.5%, *P* = 0.561) (Table 3). There was no significant difference in other adverse events such as hand-foot syndrome, decrease in platelet count, and liver dysfunction between the 2 groups. However, the DS1 group showed significantly lower incidence of degree III hand-foot syndrome than the DX group (0 vs 23.1%, *P* = 0.025).

**TABLE 3:**
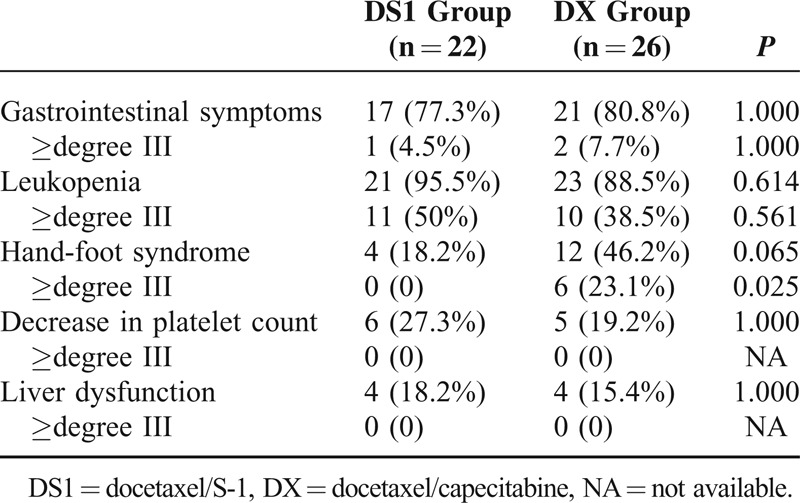
Comparison of Adverse Events Between the DS1 Group and the DX Group (n, %)

In the DS1 group, 4 patients adjusted the chemotherapy dose (18.2%, 4/22), and 1 patient discontinued the treatment because of the adverse events (4.5%, 1/22). In the DX group, 4 patients adjusted the chemotherapy dose (15.4%, 4/26), and 2 patient discontinued the treatment because of the adverse events (7.7%, 2/26). There was no significant difference in treatment changes between the 2 groups. All the adverse events were well managed after symptomatic treatment. No adverse event-associated death or severe adverse events were recorded.

## DISCUSSION

Combination chemotherapy of docetaxel and capecitabine is recommended by the National Comprehensive Cancer Network guideline for the treatment of advanced breast cancer.^12^ In comparison to the docetaxel monotherapy, the DX combination chemotherapy can significantly increase the objective response rate (42% vs 30%), TTP (6.1 vs 4.2 months), and even overall survival (14.5 vs 11.5 months).^8^ Capecitabine is a prodrug of 5-FU.^13,14^ The DX regimen has been widely used as the first-line treatment for advanced breast cancer.

S-1 has been approved for the treatment of gastric cancer, breast cancer, nonsmall cell lung cancer (NSCLC), and pancreatic cancer.^15,16^ A phase II clinical trial with 111 patients showed that S-1 monotherapy achieved a response rate of 41.7% and 1-year survival rate of 74.1% with satisfactory safety profiles.^11^ Other studies have shown that S-1 monotherapy and capecitabine monotherapy have similar efficacy in the treatment of metastatic breast cancer.^11,17,18^ As DX combination therapy has shown significantly improved efficacy in treating advanced breast cancer, we wondered whether the DS1 combination therapy can also achieve good efficacy. Although several clinical trials have shown the efficacy and safety of the DS1 regimen in treating gastric cancer and NSCLC,^19,20^ its use in advanced breast cancer has not been reported. An animal study showed that DS1 combination therapy has better efficacy than monotherapy because of the synergistic effects of decreasing the dihydropyrimidine dehydrogenase levels in tumor cells.^21^ Another phase II clinical trial also showed good efficacy of the DS1 regimen after adriamycin treatment in the neoadjuvant therapy of breast cancer.^22^

Our study found that the DS1 group and the DX group have similar TTP (median, 9.04 vs 10.94 months, *P* = 0.473). The 2 groups also showed no significant difference in objective response rate, disease control rate, and clinical benefit rate. These results suggest that first-line treatment of the DS1 regimen and the DX regimen has no significant difference in short-term efficacy in treating metastatic breast cancer. However, our study had better response rate and TTP in the DX group than the study of O'Shaughnessy et al.^8^ Our patients did not receive chemotherapy after metastasis before inclusion. In the study of O'Shaughnessy et al, only 35% patients of the DX group were treated with first-line chemotherapy, and others were receiving second- or third-line chemotherapy. In addition, our patients had higher positive rate of hormone receptors (80.8% vs 39.0%), but lower percentage of patients with ≥3 metastatic lesions (46.2% vs 64.0%) than O'Shaughnessy et al's study. These differences might explain the discrepancy between our study and O'Shaughnessy et al's study. In addition, our patients without progression after the DX regimen chemotherapy all received subsequent anticancer treatment. In the DS1 group, 11 patients received maintenance treatment of S1 monotherapy for a median time of 9.1 months (range, 5.5–12.7 months). In the DX group, 15 patients received maintenance treatment of capecitabine monotherapy for a median time of 8.6 months (range, 4.3–12.9 months). The 2 groups did not differ significantly in the median time of maintenance treatment (*P* = 0.985, HR = 0.992, 95% CI 0.433–2.273). It has been reported that patients with clinical benefits from the DX regimen would have better TTP if receiving capecitabine maintenance treatment.^23^ Therefore, our study may achieve better efficacy than other studies. At present, there are no reports on the treatment of advanced breast cancer with the DS1 regimen. However, in comparison to previous studies of advanced breast cancer treated with S-1 monotherapy, the DS1 combination chemotherapy has improved TTP and objective response rate than S-1 monotherapy.^11^

The most common adverse events were gastrointestinal symptoms and leukopenia in our study. We found no significant difference in adverse events between the DS1 group and the DX group. However, the DS1 group showed significantly lower incidence of degree III hand-foot syndrome than the DX group (0% vs 23.1%, *P* = 0.025). In 2 studies of metastatic breast cancer treated with capecitabine monotherapy, the incidence of degree III hand-foot syndrome was 21% and 22%, respectively,^17,18^ whereas the study on the metastatic breast cancer treated with S-1 reported no degree III hand-foot syndrome.^11^ These findings are consistent with our results. There are no reports on the direct comparison of adverse events between S-1 and capecitabine in the treatment of breast cancer. In the treatment of advanced gastric cancer, capecitabine showed significantly higher incidence of hand-foot syndrome than S-1.^24^ These 2 drugs are similar in other adverse events. These findings are consistent with our results. In our study, other adverse events included decreased platelet count and liver dysfunction, which did not differ significantly between the DS1 group and the DX group. Generally, all the adverse events in our study were well tolerated and well managed after symptomatic treatment. No adverse event-associated death or severe adverse events occurred in our patients.

In conclusion, this retrospective study found that first-line treatment of advanced breast cancer with the DS1 regimen or the DX regimen showed similar efficacy and safety profiles. The DS1 regimen showed significantly lower incidence of degree III hand-foot syndrome than the DX regimen. However, because of the retrospective nature and the small sample size, further prospective study with large sample size is needed to confirm the results of our study.
